# T-cell receptors that are *k*-binding have defined sequence features

**DOI:** 10.3389/fimmu.2025.1621201

**Published:** 2025-12-03

**Authors:** Hyunjin Park, Jonathan Krog, Brandon Carter, Pallavi A. Balivada, Emily M. Pogue, Samyuktha Anand, Michael E. Birnbaum, David K. Gifford

**Affiliations:** 1 Computer Science and Artificial Intelligence Laboratory, Massachusetts Institute of Technology, Cambridge, MA, United States; 2 Department of Biological Engineering, Massachusetts Institute of Technology, Cambridge, MA, United States; 3 Koch Institute for Integrative Cancer Research, Cambridge, MA, United States

**Keywords:** immunological specificity, TCR, T-cell receptor, TCR cross-reactivity, MHC class I, HLA-A allotype, HLA-A*02:01, k-binding

## Abstract

Previous studies have revealed that individual T cell receptors (TCRs) can recognize a diverse set of peptide targets displayed by Major Histocompatibility Complexes (MHCs) to enable effective adaptive immune surveillance. However, how TCR sequences encode their cross-reactivity remains poorly understood. Here, we used an *in vitro* assay to characterize the *k*-binding of 19^6^ (~47 million) different TCRs in the context of a single TCR framework for binding to seven related peptides displayed by HLA-A*02:01. We define *k-binding* to be the number of peptide-MHC targets recognized by a TCR within a specific universe of targets. We found a hierarchy of TCR complementarity-determining region 3 (CDR3) alpha and beta chain residue importance that determined *k*-binding for the seven targets. Our machine learning model that embedded TCR sequences using BLOSUM-50 provided an overall F1 score of 0.698 and an AUPRC of 0.745 for predicting TCR-pMHC binding, which was significantly superior to model results from VHSE-8 embedded or one hot encoded sequences. When we used our model to predict observed *k*-binding, we found that experimentally derived sequence motifs do not fully explain the relative importance of different CDR3 residues. We determined CDR3 residue importance by examining the reduction in machine learning model predictive ability by masking individual CDR3 residues. We found that the resulting residue importance ranking was significantly correlated to residue importance determined with a computational alanine scan using Rosetta. Our findings validate past theoretical predictions of TCR cross-reactivity and demonstrate that TCRs used in therapeutics must be carefully evaluated for their specificity.

## 1 Introduction

T cells play an important role in the adaptive immune system’s defense against pathogens and cancer. T cell receptors (TCRs) identify non-self-peptide epitopes that are presented by major histocompatibility complex molecules (MHCs) on the cell surface. Cells that display non-self-peptide epitopes on their MHCs are candidates for elimination by T-cells. As the number of potential peptide epitopes exceeds the number of TCR clonotypes contained in an individual’s T cell repertoire, a single TCR must be able to recognize a large number (~10^6^) of peptide epitopes to enable effective T cell surveillance (1).

Sewell (2) refined upon Mason’s (1) perspective and argued that TCR cross-reactivity should be understood through biophysical and structural bases, not merely through simple sequence homology. Sewell proposed multiple possible biophysical and structural mechanisms through which TCR cross-reactivity might be achieved, including altered peptide binding angle, altered peptide binding register, complementarity-determining region (CDR) loop flexibility, and residue-focused TCR engagement that tolerate certain amino acid substitutions in target peptides.

Previously, interrogation of a single TCR against multiple peptide targets or a single pMHC against multiple TCRs has been possible. Birnbaum et al. (3) selected five different human and mouse TCRs and profiled their binding of ~2 x 10^8^ pMHCs distinct pMHCs via a yeast display library. Deep sequencing of the enriched libraries after each round of iterative selection elucidated pMHCs recognized by the TCRs of interest. Their work demonstrated the structural conservation of the TCR interaction, agreeing with Sewell’s insight. Meanwhile, NetTCR-2.0 (4), a convolutional neural network (CNN)-based model trained on publicly available bulk CDR3β-pMHC binding data, predicts the binding of a TCR sequence against three target peptide epitopes (GILGFVFTL, NLVPMVATV, and GLCTLVAML), although the accuracy of their model was hindered by the overall low quality of the publicly available datasets.

**Figure 1:**
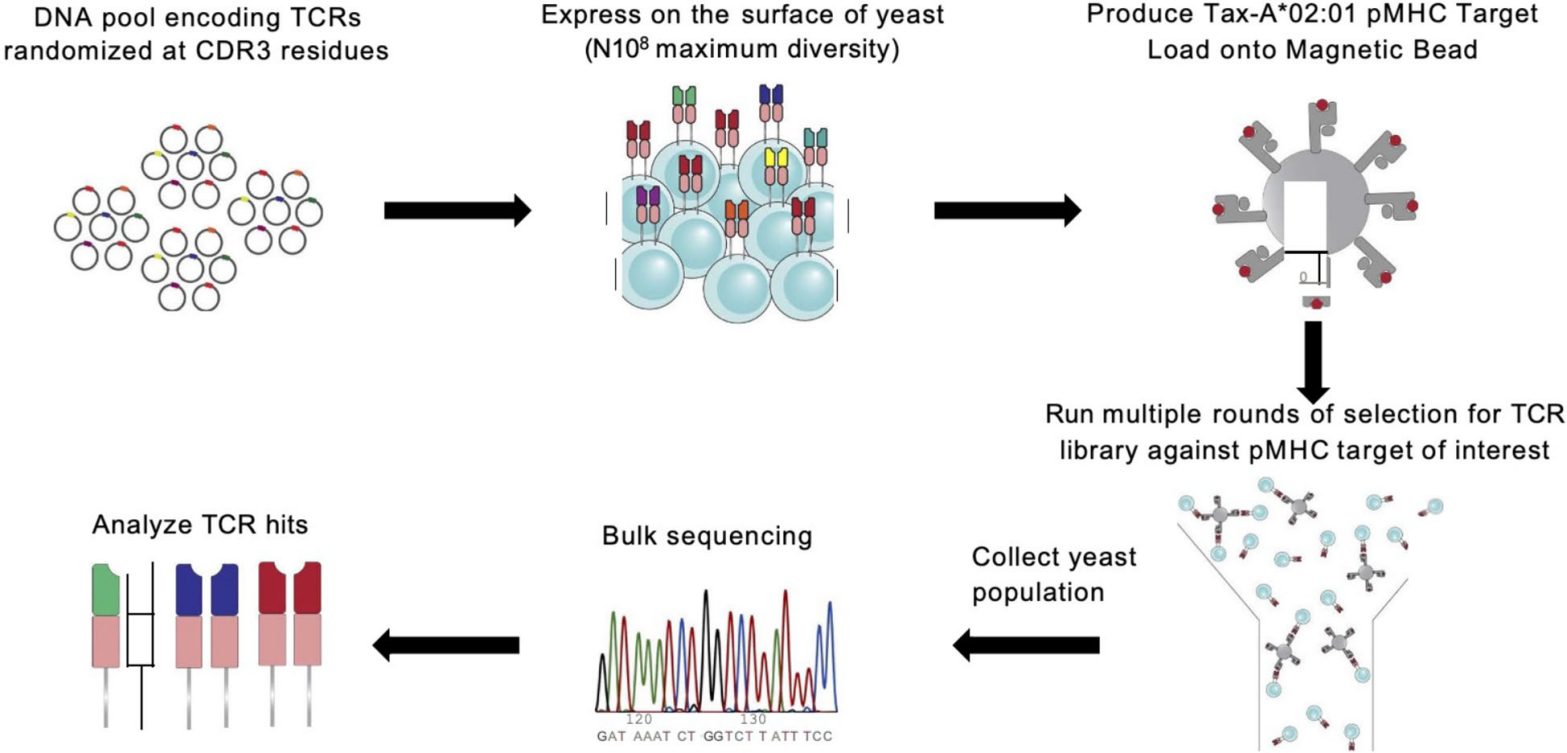
A6 TCR is randomized and panned against HLA:A*0201 presenting seven peptide epitopes. Overview of experimental protocol for generating TCR-pMHC binding data with yeast display selection via MACS (3, 22, 23).

**Figure 2:**
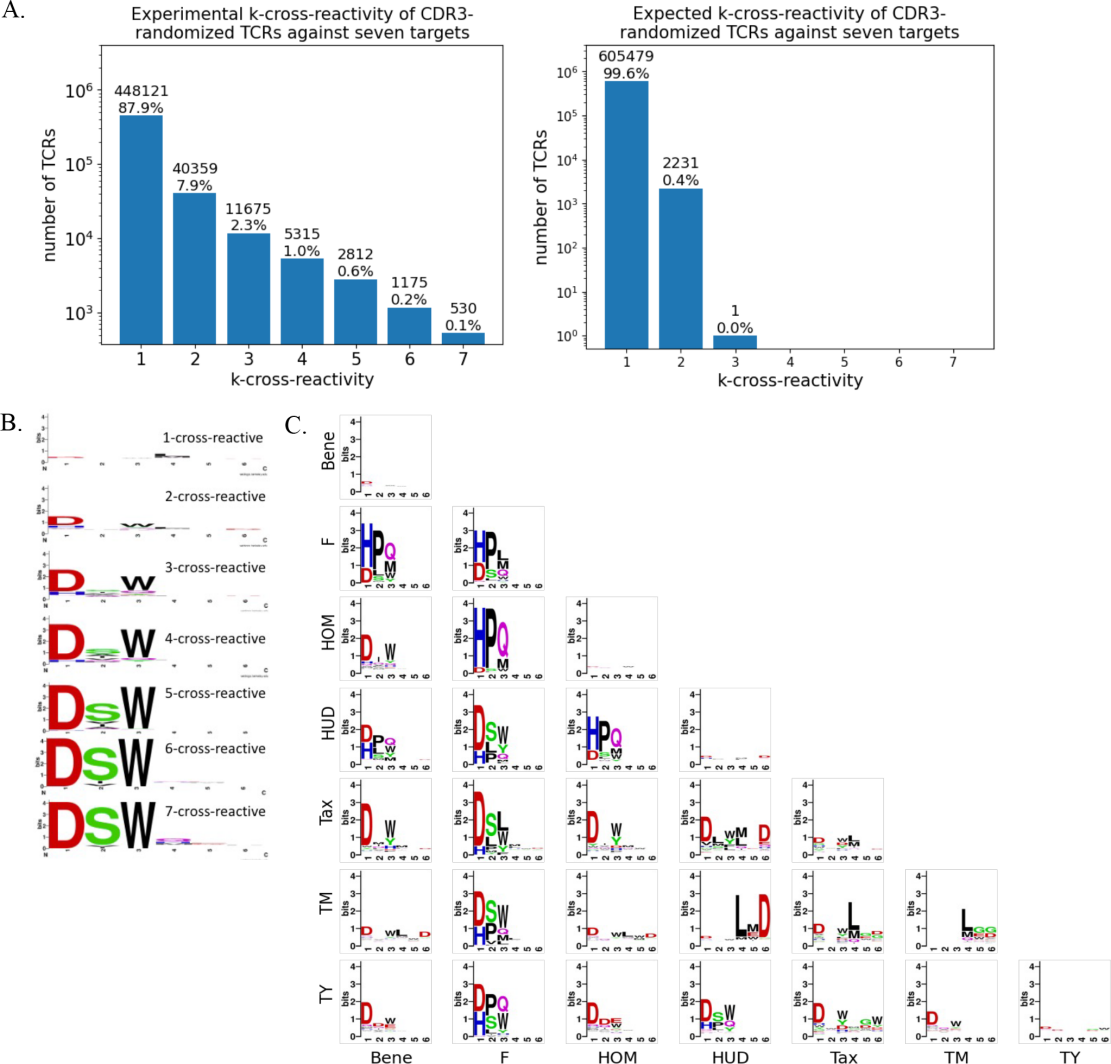
BLOSUM50 embedding improves TCR binding classification. We trained MLP models that take TCR sequences embedded in different sequence embeddings and output binding prediction against the seven peptide targets. As the weights are shared internally, the models implicitly learn to predict cross-reactivity. We performed 10-fold cross-validation for the MLP models. The differences in predictive performances of the models trained using different sequence embeddings were measured for different cross-reactivity strata and peptide targets. **(A)** Models trained with BLOSUM50 embedding outperformed those with VHSE8, which in turn outperformed one-hot (∀metrics, replicates *p<* 0.001; Kruskal-Wallis test followed by Dunn’s *post-hoc* test, FDR correction). Contrastingly, the performances of the models trained with the same embedding did not differ significantly (*p* > 0.05). **(B)** The degrees of performance improvement were significantly varied for different cross-reactivity strata (*p<* 0.0001, *ϵ^2^* = 0.73). **(C)** Further, the degrees of improvement were significantly varied for different pMHC targets (*p*< 0.0001, *ϵ^2^* = 0.85).

**Figure 3:**
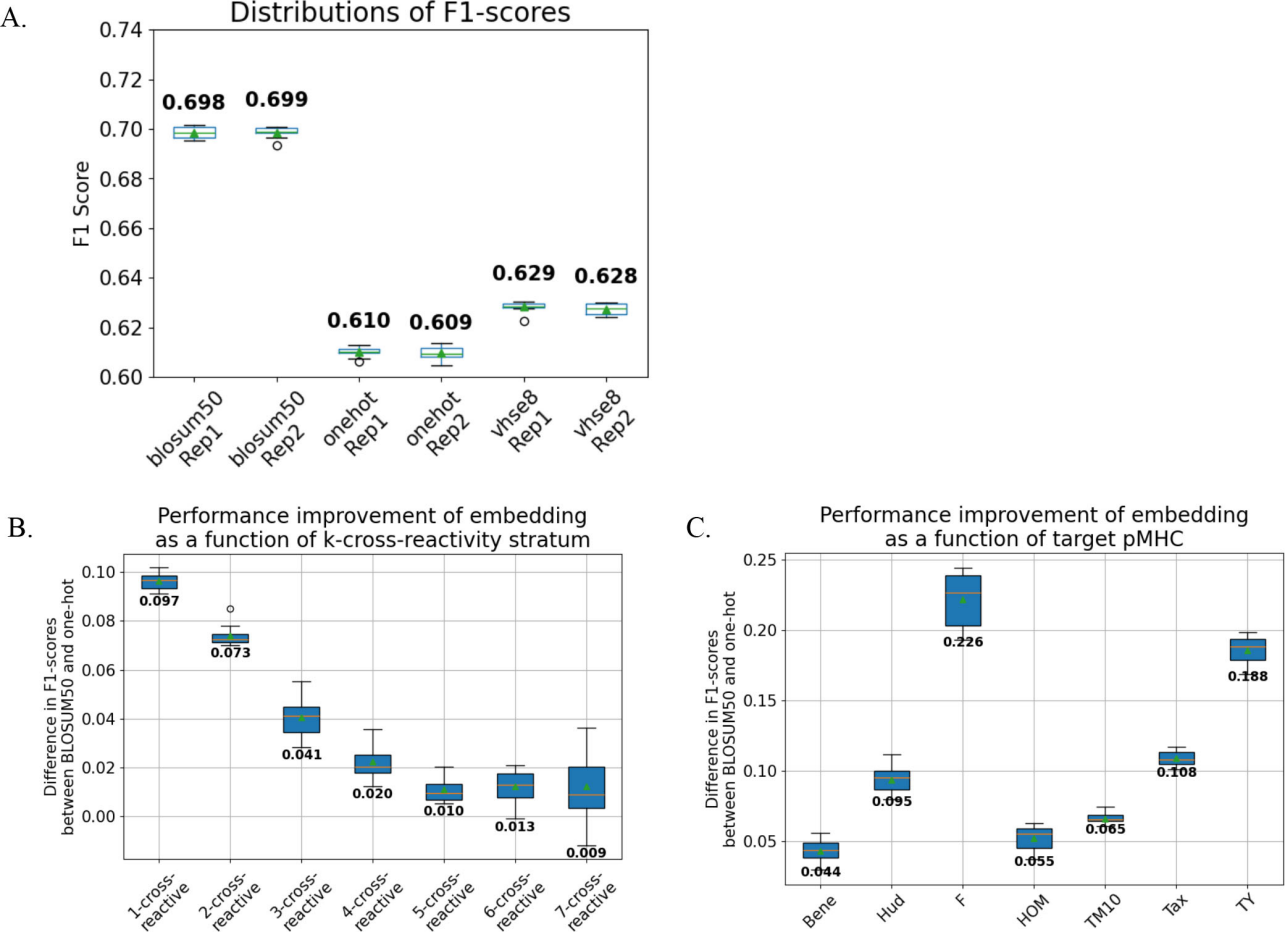
Sequence-masked training shows the hierarchical contribution of each amino acid position. We performed sequence-masked training in which different residue positions are deleted to estimate the marginal contribution of each residue. **(A)** While masking any residue resulted in a significant deterioration (*p* < 0.001, one-sample *T*-test), the degrees of deterioration were significantly varied for different positions being masked (*p<* 0.0001, *ϵ^2^* = 0.21). The inferred hierarchy of marginal importance for different residue positions was: a99 > a101 > b98 > a100 > b100 > b99. Further, while masking either of CDR3 chain caused significant performance deterioration, masking α chain caused significantly greater deterioration than masking β chain (*p<* 0.001, Wilcoxon signed-rank test). **(B)** We performed computational alanine substitution and used FlexPepDock (13, 14) to infer the changes in interface energy (*ΔI_sc*, lower more stable) following alanine substitution. We found that *ΔI_sc* explains the ML performance deterioration, as measured by *ΔF1 score*, following residue and chain masking.

To refine the frameworks by Mason (1) and Sewell (2), we determined how individual CDR3 residues contribute to cross-reactivity. We trained our ML models on densely sampled, randomized A6 TCR CDR3α and CDR3β sequences (**Table 1**) screened against seven target peptides (**Table 2**) presented by HLA-A*02:01 (**Figure 1**; *Methods*). We first demonstrate that TCR sequences exhibit a high level of cross-reactivity across the selected peptide sequences. Second, we demonstrate that the ML models provided with biologically informative embeddings – BLOSUM50 (5) and VHSE8 (6) – significantly improve TCR binding prediction over the ML models endowed only with sequence information, thereby computationally validating Sewell’s (2) proposal that structural and biophysical properties influence cross-reactivity (**Figure 2**). Finally, we establish a link between TCR binding and change in interface energy following residue-level substitutions, thereby computationally demonstrating how certain TCR residues differentially influence pMHC recognition by TCRs, confirming the structural and thermodynamic underpinnings of TCR cross-reactivity (**Figure 3**).

**Table 1:** Modified residues on CDR3 alpha and beta chains of A6 TCR.

CDR3 Alpha Beta Library	
Randomized Positions	Alpha 99-101, Beta 98-100
Native Sequence	DSW/LAG
Estimated Diversity	19^6^

The amino acid coordinates for randomized positions follow the definitions provided by Smith et al. (24).

**Table 2:** List of peptide sequences and their constituent amino acid sequences.

Assayed Peptides									
Peptide Name	P1	P2	P3	P4	P5	P6	P7	P8	P9
HTLV-1 Tax-1 peptide (Tax)	L	L	F	G	Y	P	V	Y	V
ELAV-like protein 4 (HUD)	L	G	Y	G	F	V	N	Y	I
BENE	L	L	Q	G	W	V	M	Y	V
Phosphofructokinase (F)	T	M	G	G	Y	C	G	Y	L
Tyrosine Kinase (TY)	S	L	H	G	Y	K	K	Y	L
Tax TM10 (TM)	T	L	W	G	W	V	K	Y	V
Homeobox (HOM)	N	L	Q	G	S	P	V	Y	V

A previous work indicated that these peptides activate the native (DSW/LAG) A6 TCR in an *in vitro* cell lysis study (19).

In sum, we show that TCRs are highly cross-reactive, and ML models can effectively predict their cross-reactivity. ML models can achieve low computational cost when provided with relevant biophysical information via *a priori* sequence embeddings, thus enabling them to be applied to high-throughput deep sequencing datasets of TCR-pMHC pairs even when high-resolution crystal structures of individual TCR-pMHC pairs are not available. Our work serves as the groundwork for the many-to-many interrogation of TCR-pMHC interactions using a sequence-based ML model, a step towards the mechanistic understanding of the TCR cross-reactivity.

## 2 Materials and methods

### 2.1 Creation of TCR yeast display libraries and pMHC monomers

In this study, we generated a novel experimental dataset linking TCR-pMHC binding pairs. We selected the A6 T cell receptor as our model framework based on its well-characterized interaction with the native ligand Tax-HLA*A:0201. This framework sequence was expressed as a single-chain TCR (scTv) following the format described by Aggen et al. (7), using the pCT302 expression vector and the Aga2 leader peptide.

To diversify this framework, we targeted residues in the CDR3ɑ and CDR3β regions for randomization guided by their proximity to the peptide in the previously solved TCR-pMHC crystal structure. This analysis resulted in the creation of our TCR library with 3 positions in the CDR3ɑ (residues 97-100) and CDR3β (residues 98-100) being fully randomized in amino acid space generating a TCR library with an estimated diversity of 4.7 x 10^7^ unique variants.

The pMHC reagents were expressed in single-chain trimer format as previously described by Hansen et al. (8), with components linked by GS linkers in the following order: signal peptide for excretion, peptide, human b2m, HLA:A*0201, AviTag (for biotinylation) and 8x His (for purification). This construct was cloned into the conventional pVL1393 insect cell expression plasmid and transfected into SF9 insect cells for production. All seven pMHC variants were expressed in this manner and used for downstream MACS selections.

### 2.2 Yeast display library selections and sequencing

To identify TCR-pMHC binding pairs, we performed independent selection campaigns against each of the seven pMHC targets using the TCR library. Each selection consisted of three consecutive rounds of magnetic-activated cell sorting (MACS) with streptavidin (SAV) microbeads. For each round, yeast expressing the scTv constructs were induced for surface expression, and an input population representing 10× coverage of the library’s theoretical diversity was prepared (e.g., for a 5 × 10⁷ library, at least 5 × 10^8^ yeast cells were used).

MACS selections were performed using 250 µL of SAV microbeads pre-loaded with pMHC at a concentration of 400 nM. The yeast and pMHC-coated beads were incubated together for 2–3 hours at 4 °C with gentle rotation. The mixture was then passed through magnetic capture columns, and bead-bound yeast were retained and eluted. This enriched population was expanded to full density and used as input for the next round of selection. This iterative process was repeated three times for each pMHC target, with biological replicates conducted for all seven targets.

Following each round, 250 µL of the selected yeast population was collected for DNA extraction. TCR-encoding regions were PCR-amplified, during which Illumina sequencing adapters were added. The resulting libraries were submitted to the MIT BioMicro Center and sequenced on an Illumina NovaSeq 6000 using a high-throughput, paired-end multiplexed format.

### 2.3 Creation of logos for TCR sequences with different target specificities

**Figure 4:**
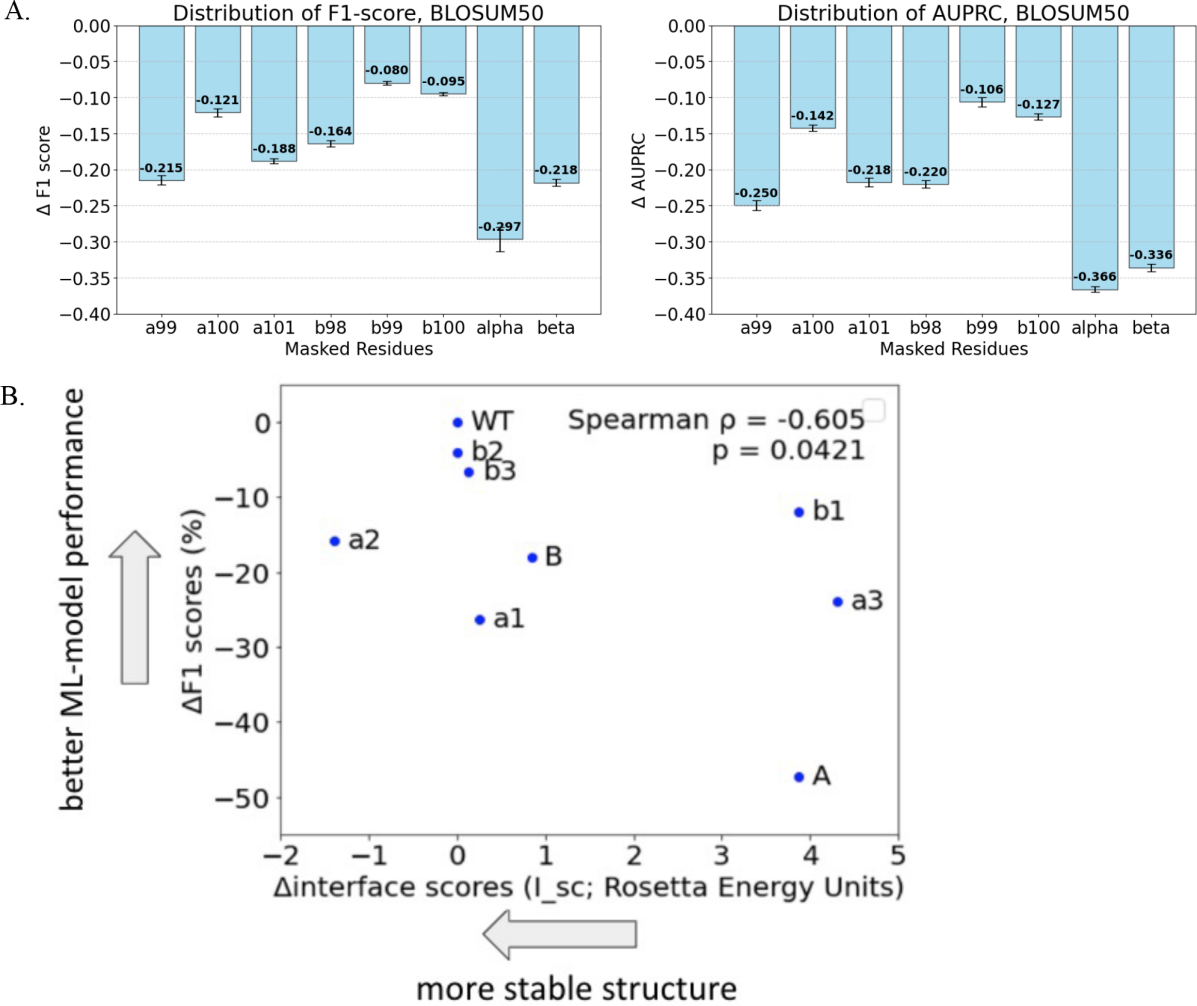
TCRs are highly cross-reactive. **(A)** CDR3 sequences binding to one or more of examined pMHC targets are more cross-reactive than expected by chance (p ≈ 0.0; one-sided binomial test). **(B)** Cross-reactive CDR3 sequences contain learnable sequence features enriched over target-specific sequences. **(C)** The logos of 2-binding CDR3 sequences (off-diagonal) contain richer features than the logos of 1-binding sequences (target-specific; diagonal), suggesting that the cross-reactivity cannot be predicted only from binding of individual peptides. The logos are generated using WebLogo (9).

We refer to the set of seven peptides listed in **Figure 1C** as the Set U. We refer to a TCR to be “S-binding” for the set S of peptides if the TCR binds to the peptides in S and does not bind to the peptides in U-S. We call a TCR to be “*k*-binding” if the TCR is S-binding and *k* is the size of the Set S. We defined the list of *k*-binding TCR sequences from *k* = 1 to *k* = 7 and created the sequence logo for each *k* (**Figure 4B**) using WebLogo (9).

To examine the diversity of 1- and 2-binding TCR sequence, we defined the list of S-binding TCR sequences for *k* = 1 and *k* = 2 for all possible S. The resulting 28 logos, 7 for 1-binding (i.e., target-specific) and 21 for 2-binding TCR sequences, were created using WebLogo (**Figure 4C**).

### 2.4 Design and training of machine learning models

We trained multi-layer perceptron (MLP) models to predict binding against seven peptide targets listed in **Table 2**. The models take TCR CDR3α/β sequences encoded using three different embeddings: BLOSUM50 (5), VHSE8 (6), and one-hot encoding. Since the length of each input TCR sequence is 6, BLOSUM50 and one-hot encodings map each amino acid to 20-dimensional vector, and VHSE8 encoding maps each amino acid to 8-dimensional vector, the input dimensions were 120 (= 6*20) for BLOSUM50 and one-hot encodings and 48 (= 6*8) for VHSE8 embedding.

Each neural network contains two hidden linear layers with 500 neurons and rectified linear unit (ReLU) activation functions. The output layer returns 7-dimensional vectors passed through a sigmoid layer, each entry representing the binding probability of the TCR against each of 7 peptide targets presented by HLA-A*02:01 (**Table 2**). The cross-entropy loss was minimized via Adam optimizer (10) with the learning rate parameter of 0.0001 and the weight decay parameter of 0.0005. All models are trained for 10 epochs with a batch size of 1028.

Non-binding TCRs outnumber binding TCRs for all target peptides. To overcome this intrinsic class-imbalance in our datasets, we employed a weighted sampling strategy based on *k*-binding. First, TCR sequences are stratified according to their *k*-binding. Next, for each cross-reactivity stratum (value of *k*), we computed a weight that is inversely proportional to the frequency of the stratum. Consequently, the TCR sequences with higher *k*-binding had greater weights. Formally,


$$w_{k}=sqrt\left(n_{0}/n_{k}\right)$$

where $n_{k}$ is the number of *k-*binding TCR sequences. Third, the weights $w_{k}$ are used to sample TCR sequences with replacement during training.

### 2.5 Validation and evaluation of machine learning models

We used 10-fold cross-validation of the training and validation sets (9:1 split) in two independent replicates with different random seeds. We confirmed the model performances to be robust against the random initialization via different random seeds (**Figure 2A**, **Supplementary Figure 4A**).

All models are evaluated using F1-score and Area Under the Precision-Recall Curve (AUPRC) metrics; these metrics were chosen over more commonly used metrics of accuracy and Area Under the Receiver Operating Characteristic Curve (AUROC) due to their robustness against class-imbalanced datasets. For the detailed comparison of performances of models trained with different sequencing embeddings, we (i) stratified the sequences according to their *k*-binding and (ii) according to their bound target peptides and computed F1-scores and AUPRC for stratified sequence sets (**Figures 2B, C**, **Supplementary Figures 4B, C**). The statistical significance of model performance differences in different cross-reactivity strata and target peptides was tested using Kruskal-Wallis test, and ϵ^2^ was computed to measure their effect size, defined as the proportion of variance explained by group differences (11).

We benchmarked the performance of our MLP models against the logistic regression classifiers trained independently per peptide target and permuted MLP models where the relationships between TCR sequences and peptide targets of the training datasets were broken by random shuffling (**Supplementary Figure 3**). All models were trained with BLOSUM50-encoded TCR sequences. The MLP models, which could leverage shared internal weights to simultaneously predict binding across multiple target peptides, significantly outperformed logistic regression models (*p* = 6.0e-06, Mann-Whitney *U*-test). The outperformance of the MLP models persisted across all cross-reactivity strata (*p* = 0.0020, Wilcoxon signed-rank test) and all individual target peptides (*p* = 0.0020). Note that the most pronounced performance improvement occurred within the 7-binding stratum, demonstrating the capability of the MLP model to implicitly learn the sequence feature representation of cross-reactivity in a supervised manner.

### 2.6 Masked training of machine learning models

We created synthetic datasets for residue-masked training and validation by deleting the residue(s) to be masked from the original datasets with all six residues. The models were not provided with information on *which* residue positions are deleted. Note that in the residue-masked datasets the same sequence can be mapped to two different sets of bound target peptides.

The total of eight synthetic datasets were created: masking of residues 1 through 6, of α chain, and of β chain. The MLP model architecture is edited appropriately for each synthetic dataset so that the number of dimensions in input dataset elements accords with the dimensionalities of synthetic datasets transformed with each sequence embedding.

The 10-fold validations were performed, and the models were evaluated using F1-score and AUPRC metrics for different cross-reactivity strata and target peptides as was the case for the models endowed with all six residues. The differences in performances between the models trained with masked synthetic datasets and those trained with full datasets were measured using the differences (Δ) in F1-scores and AUPRC metrics for different cross-reactivity strata and target peptides (**Figure 3A**, **Supplementary Figure 5A**). The statistical significance of performance differences of models trained using synthetic datasets with different residue being masked was tested using Kruskal-Wallis test, and ϵ^2^ is computed to measure their effect size, the proportion of variance explained by group differences (11). The statistical significance of performance differences of models trained using synthetic datasets with masked α vs. β chain was tested using Wilcoxon signed-rank test (12).

### 2.7 Use of FlexPepDock to infer interface binding energy

To infer interface binding energy, we used FlexPepDock (13, 14) to infer interface binding energy between TCR and pMHC. To generate input PDB files, we downloaded the PDB file of structure 1AO7 (15) from RCSB Protein Data Bank (16). We standardized the PDB file as recommended by PyRosetta (17) and reordered the order of chains such that TCR chains appear before the peptide chains in the PDB file. This is the wildtype (WT) PDB file.

To perform computational alanine scanning, the appropriate amino acid in the wildtype (WT) PDB file is replaced by alanine. This is achieved by updating the amino acid identity (e.g. from “D” to “A”) as well as substituting the atoms pertaining to the original amino acid to the atoms pertaining to alanine. The total of eight substituted PDB files were created: alanine substitution of residues 1 through 6, of all three α chain residues, and of all three β chain residues. The short- and long-form nomenclatures of corresponding PDB files are [a1, a2, a3, b1, b2, b3, A, B] and [a99, a100, a101, b98, b99, b100, alpha, beta] (**Figure 3B**, **Supplementary Figure 5B**).

Nine FlexPepDock jobs – one for WT and eight for each substituted PDB file – were undertaken. For each run, one hundred high-resolution structures were generated with receptor backbone minimization; no structure was generated with low resolution preoptimization protocol. We computed the median interface scores (*I_sc*) of one hundred FlexPepDock structures. We correlated the difference between the median interface scores between WT and alanine-substituted structures (Δ *I_sc*) with the ML performance changes due to residue masking (*ΔF1 score* and *ΔAUPRC*) as measured above and computed its size and statistical significance via Spearman’s rank correlation (**Figure 3B**, **Supplementary Figure 5B**).

## 3 Results

### 3.1 Screening of randomized CDR3 TCRs against HLA-A*02:01 presenting seven peptide epitopes

We focused on the A6 TCR, a well characterized model system TCR originally derived from an HTLV-1-infected patient that recognizes the Tax peptide derived from HTLV (LLFGYPVYV) in the context of HLA-A*02:01 (18). Through years of study, this TCR has multiple described peptide mimotopes with a range of sequence divergence from the cognate Tax peptide. Using the X-ray crystal structure of A6 TCR binding to Tax presented by HLA-A*02:01 (15), we selected six residues within A6 TCR complementarity-determining regions 3 (CDR3) α and β chains that were the closest molecular contacts to the peptide (**Figure 1**). We screened the resulting library against seven of the peptides reported to be cross-reactive with A6 (19) presented by HLA-A*02:01 through three yeast-display panning rounds (3). These seven peptides set a suitable stage to systematically quantify *k*-binding.

To understand if our selection campaign is in fact enriching for high-fidelity binders against each target, we first quantified the number of unique TCR sequences observed in each panning round (**Supplementary Figure 1A**). We observed that the number of unique TCR sequences decrease across all target peptides and replicates with the progression of the panning rounds as the TCR sequences with weak to no affinity against the given peptide target are dropped out. Second, we quantified the percentages of reads contributed by the top 100 TCR sequences (**Supplementary Figure 1B**). We observed that the percentages of the top 100 TCR sequences increase across all target peptides and replicates with the progression of the panning rounds as these sequences with strong affinity against the given peptide target are enriched. Finally, to visually inspect the enrichment of amino acid preference at each residue position and convergence of two replicates during panning rounds, we performed weighted sampling (*Methods*) of the TCR sequences in each round of panning against the *Tax* peptide and created sequence logos (**Supplementary Figure 1C**). We confirmed not only that certain amino acid preferences are enriched in position-dependent manner, but also that these preferences in two replicates are convergent.

To validate the yeast-display selection results we conducted a titration assay on a subset of selected TCRs with varying predicted cross-reactivities (**Supplementary Figure 2**). Eight TCR clones, both containing and lacking the “DSW” motif identified during yeast selections, were individually expressed on yeast and stained with pMHC tetramers at concentrations ranging from 0.1 nM - 200 nM (**Supplementary Methods**). The resulting titration curves provided quantitative confirmation of TCR affinity predictions derived from yeast panning (*Methods*).

We found that yeast-display selection enriched all binders identified by the titration assay (**Supplementary Figures 2C, D**). The few binders identified by yeast-display but not by titration assay (such as for the HUD peptide), can be explained by the higher effective avidity of yeast-display bead-based selections than titration soluble staining reagents, which has been previously seen to identify binders that may not be observed with pMHC-TCR interactions measured via tetramers (3). Overall, our titration assay data reinforces the reliability and biological relevance of our high-throughput selection data.

### 3.2 TCRs exhibit extensive cross-reactivity

We characterized the binding profiles of 19^6^ (approximately 47 million) randomized A6 TCR variants. The yeast panning revealed extensive cross-reactivity (**Figure 4A**). The TCR sequences present in the MACS Round 3 in both replicates were defined as binding to the given pMHC (*Methods*). Among the TCR sequences binding to at least one peptide, 448,121 sequences specifically recognized a single target peptide (1-binding), 40,359 sequences recognized two target peptides (2-binding), and 530 sequences recognized all seven peptides (7-binding). This observed cross-reactivity significantly exceeded expectations based on the assumption of random binding (p ≈ 0.0; one-sided binomial test), agreeing with the previous theoretical predictions of TCR cross-reactivity (1, 2).

Further analysis demonstrated that cross-reactive CDR3α/β sequences contained distinct sequence features, specifically the “DSW” motif in the three α chain positions, when compared to those specific to single targets (**Figure 4B**). The logos of 2-binding sequences in off-diagonal entries presented richer sequence information compared to logos derived from target-specific sequences in diagonal entries (**Figure 4C**), showing that TCR cross-reactivity could not be inferred solely from individual peptide binding events.

### 3.3 Sequence embedding methods enhance TCR binding prediction

We trained a multi-layer perceptron (MLP) model to predict TCR binding to the seven peptide-major histocompatibility (pMHC) complexes. The model inputs encoded TCR sequences, passes the encoded TCR sequences through a first hidden layer with 500 ReLU-activated neuron outputs, into a second hidden layer with 500 ReLU-activated neuron outputs, followed by an output layer that transforms its 500 neuron input into 7 outputs that are used to compute sigmoid-activated binding probabilities for all seven peptide-MHC targets. TCR sequences were encoded with three different embeddings: BLOSUM50 (5), VHSE8 (6), and one-hot encoding. Model performance was assessed using 10-fold cross-validation (*Methods*).

The models trained with BLOSUM50-embedded input sequences consistently yielded superior predictive performance (as measured by F1-score and AUPRC metrics) compared to those trained with VHSE8 or one-hot embeddings across all peptide targets and cross-reactivity strata (**Figure 2A**, **Supplementary Figure 4A**; *p* < 0.001; Kruskal-Wallis test (11) with Dunn’s *post-hoc* test (20), FDR corrected). Notably, model performance gains varied significantly according to cross-reactivity strata (**Figure 2B**, **Supplementary Figure 4B**; *p* < 0.0001, ϵ² = 0.73) and target peptides (**Figure 2C**, **Supplementary Figure 4C**; *p* < 0.0001, ϵ² = 0.85), showing that the evolutionary and biophysical information innate in biologically informative sequence embeddings provide context-sensitive information to the ML models in inferring TCR cross-reactivity.

To explicitly validate the advantages of the MLP architecture, we benchmarked the performance of our MLP models against the logistic regression classifiers trained independently for each peptide target (**Supplementary Figure 3**). Both models were trained with BLOSUM-50 encoded TCR sequences. In addition, we conducted permutation test by shuffling the relationships between TCR sequences and peptide targets of the training datasets for each cross-validation fold (**Figure 2A**), whilst leaving the test sets intact, and re-training the MLP models. Both logistic regression and permuted models utilized BLOSUM-50 encoded TCR sequences, ensuring a direct comparison.

The MLP models, which leverage shared internal weights to simultaneously predict binding across multiple target peptides, significantly outperformed logistic regression models (*p* = 6.0e-06, Mann-Whitney *U*-test) and permuted models (*p* = 3.4e-08). The superior performance of the MLP models persisted across all cross-reactivity strata (*p* = 0.0020, Wilcoxon signed-rank test) and all individual target peptides (*p* = 0.0020). Notably, the most pronounced performance improvement occurred within the 7-binding stratum, demonstrating the capability of the MLP model to implicitly learn the sequence feature representation of cross-reactivity in a supervised manner (**Supplementary Figure 3B**).

### 3.4 Contributions of individual CDR3 residues are hierarchical

We evaluated the marginal contribution of individual residues in TCR CDR3 sequences by training MLP models on sequences with specific residue positions deleted (**Figure 3A**, **Supplementary Figure 5A**). Masking any residue significantly reduced predictive performance (*p<* 0.001, one-sample *T*-test), but the magnitude of performance deterioration differed significantly among different residue positions being masked (*p* < 0.0001, ϵ² = 0.21). The marginal importance of six residues ranked as α99 > α101 > β98 > α100 > β100 > β99 regardless of sequence embeddings and performance measures. Furthermore, masking three residues on the α chain resulted in greater performance reductions compared to masking the β chain residues (*p<* 0.001, Wilcoxon signed-rank test), highlighting the dominant role of the α chain in mediating TCR recognition specificity against the related peptide targets.

To validate our observations in the light of structural information, we performed computational alanine substitutions at each appropriate residue position of the A6 TCR/Tax-HLA-A*02:01 crystal structure ( (15), PDB entry 1AO7) and used Rosetta-based FlexPepDock (13, 14) to estimate changes in interface energy (*ΔI_sc*) between the A6 TCR and *Tax* peptide (**Figure 3B**, **Supplementary Figure 5**). Computational alanine scanning closely mirrored experimental observations, with *ΔI_sc* strongly correlating with model performance deterioration measured as *ΔF1 score* and *ΔAUPRC* metrics. This result corroborates our sequence-based machine learning analyses with physics-based structural modelling.

## 4 Discussion

We found a strict hierarchy of TCR complementarity-determining region 3 (CDR3) α and β chain residue importance that determined *k*-binding for the seven targets we considered by systematically characterizing T-cell receptor (TCR) cross-reactivity with *in vitro* and computational approaches. Building on Mason’s (1) hypothesis that extensive TCR cross-reactivity is biophysically necessary, and Sewell’s (2) proposals that TCR cross-reactivity is not random but achieved via various structural and biophysical mechanisms, we confirmed experimentally and computationally that TCR sequences are indeed highly cross-reactive.

Our datasets randomized both the TCR CDR3α and β chain residues simultaneously, while only the β chain is typically randomized in most publicly available datasets (21). Furthermore, our seven target peptides were immunologically dense, all known to engage the A6 TCR. We were thus able to interrogate the intricate relationship between CDR3 residues and peptide specificity, uncovering the significance of the α chain residues – surpassing that of the β chain residues – in determining the TCR binding among the immunologically related target peptides.

Machine learning (ML) analyses revealed that biologically informative embeddings (BLOSUM50 and VHSE8), encoding evolutionary and physicochemical features, substantially improved binding predictions compared to one-hot encoded sequence information alone. This supports Sewell’s (2) framework that structural and biophysical constraints, not just sequence homology, govern TCR recognition. Moreover, our residue-masked training demonstrated that specific CDR3 residues contribute disproportionately to TCR binding, corroborated by the interface binding energy computed from structures generated by the Rosetta-based method FlexPepDock (13, 14). These findings computationally demonstrate the structural and energetic basis of TCR cross-reactivity.

Our study provides insights into TCR cross-reactivity within a focused set of related peptides presented by HLA-A02:01. However, generalizing our findings to estimate TCR cross-reactivity across the entire HLA-A02:01-restricted peptide space remains speculative. The observed strict hierarchy of CDR3 residue importance implies that highly cross-reactive TCRs exhibit fewer degrees of freedom in sequence composition compared to low cross-reactive TCRs. This observation suggests a distribution in which a relatively small number of TCR sequences recognize a very large number of peptides, whereas a larger number of TCR sequences bind to fewer peptides. Consequently, the relevant question may not be how many peptides an individual TCR can recognize – a perennial question by Mason (1) and Sewell (2) – but rather what the distribution of *k*-cross-reactivity might look like across the TCR repertoire. Future studies, using expanded peptide libraries and comprehensive modeling, should seek to characterize this distribution more explicitly to better understand the overall landscape of TCR recognition.

## Data Availability

The raw data supporting the conclusions of this article will be made available by the authors, without undue reservation.
