# Supplementary material for: T-cell receptors that are k-binding have defined sequence features
Source: Front Immunol. 2025 Dec 3;16:1621201. doi: 10.3389/fimmu.2025.1621201 (PMC12709673; doi:10.3389/fimmu.2025.1621201)
Supplement: Supplementary file 1 [file Supplementaryfile1.pdf]

Extended Data Figure 1. Sequential panning rounds implement positive selection of TCR CDR3α/β sequences

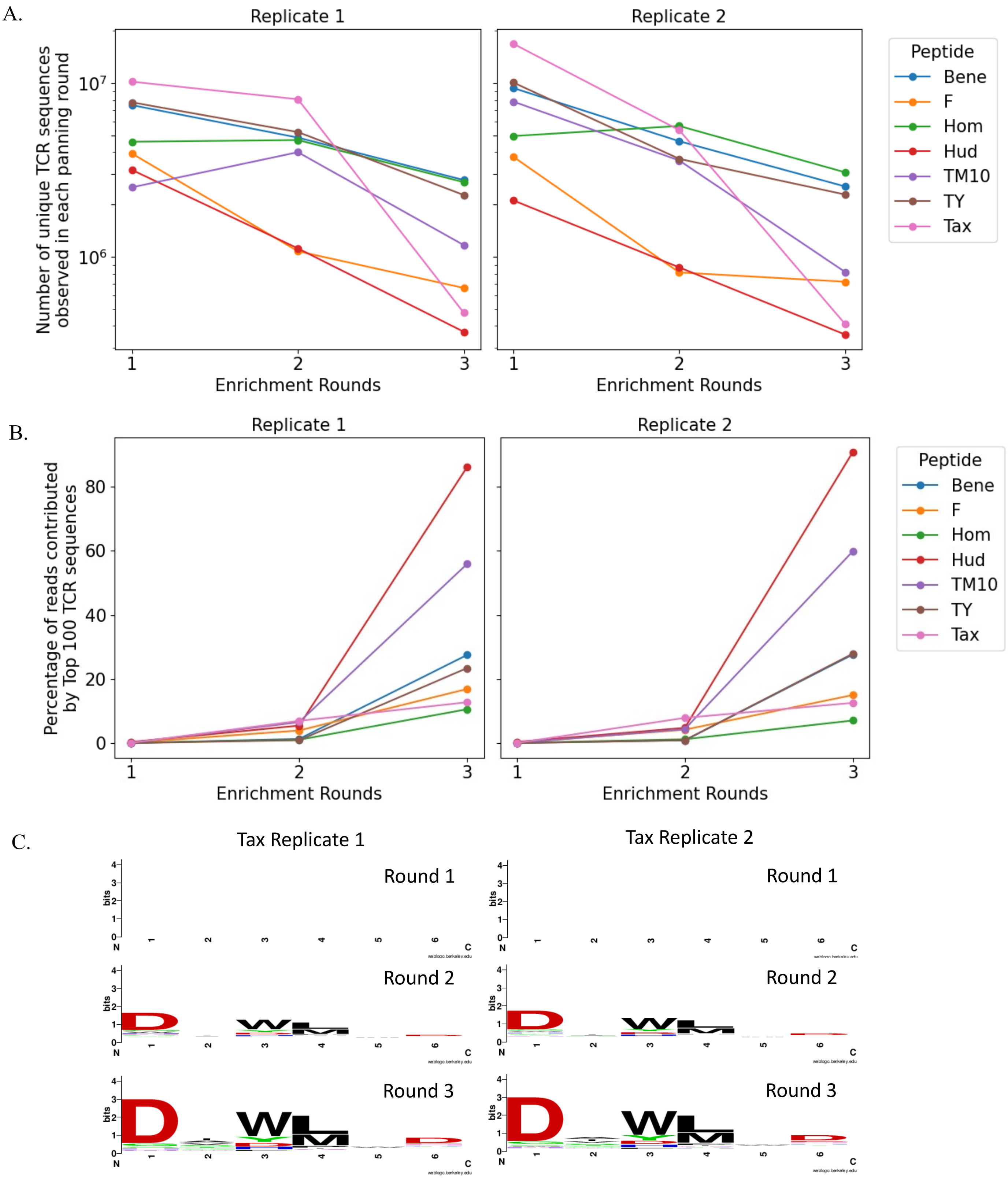

Extended Data Figure 2. Titration with pMHC monomers recapitulate the yeast panning assays.

A.

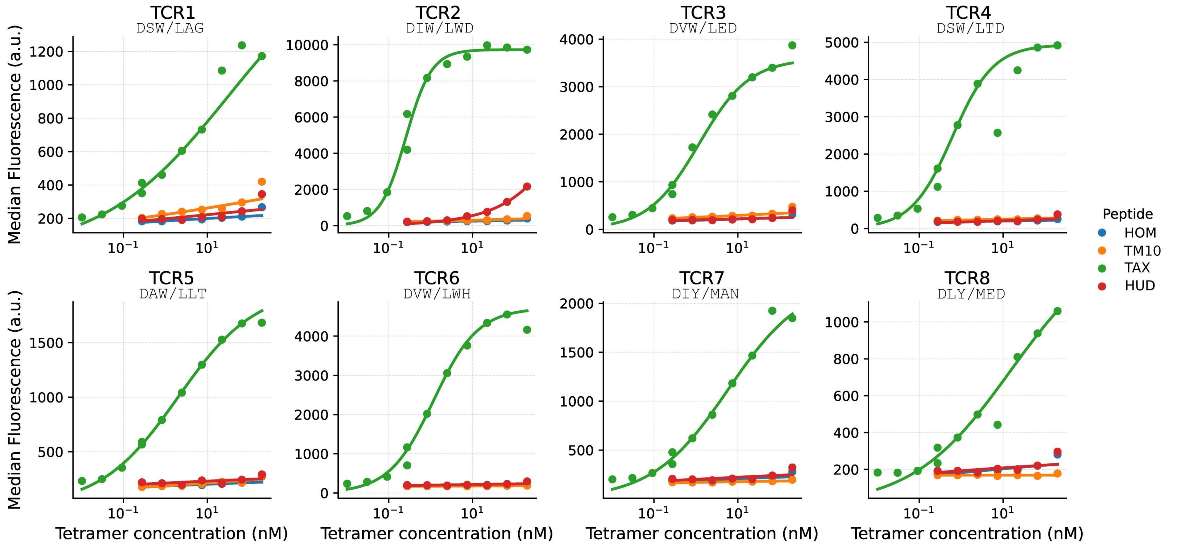

B.

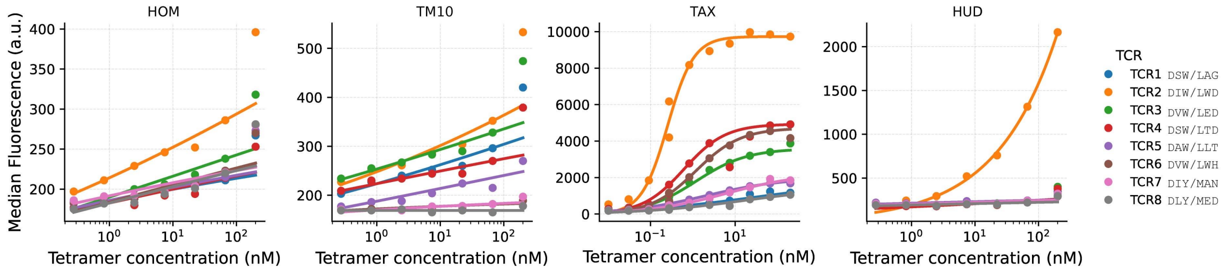

C.

TCR	CDR3a/b	BENE	F	HOM	HUD	TAX	TM10	TY
1	DSW/LAG	103	18459	125	24905	28293	140287	71
2	DIW/LWD	867119	54	1025475	79976	116306	325119	1099
3	DVW/LED	75938	654	308	1199916	101991	509142	266
4	DSW/LTD	85404	181	476	6126	108327	713281	382
5	DAW/LLT	40	0	24	0	451061	51353	25
6	DVW/LWH	27	0	1	0	129202	11	4
7	DIY/MAN	25	1	0	0	196753	1	0
8	DLY/MED	236	1352	3	2522097	229871	16	1

Binding Based on Titration:	Not determined	Binding	Subtle Binding or No Binding

D.

Replicate Combination Method	R (threshold)	*P*-value
Average (mean)	1	0.066
Average (mean)	10	0.0058
Average (mean)	100	0.00030
Average (mean)	1000	6.9e-06
Average (mean)	10000	1.4e-06
Minimum	1	0.013

Extended Data Figure 3. MLP models with shared internal weights outperform logistic regression classifiers and permuted models

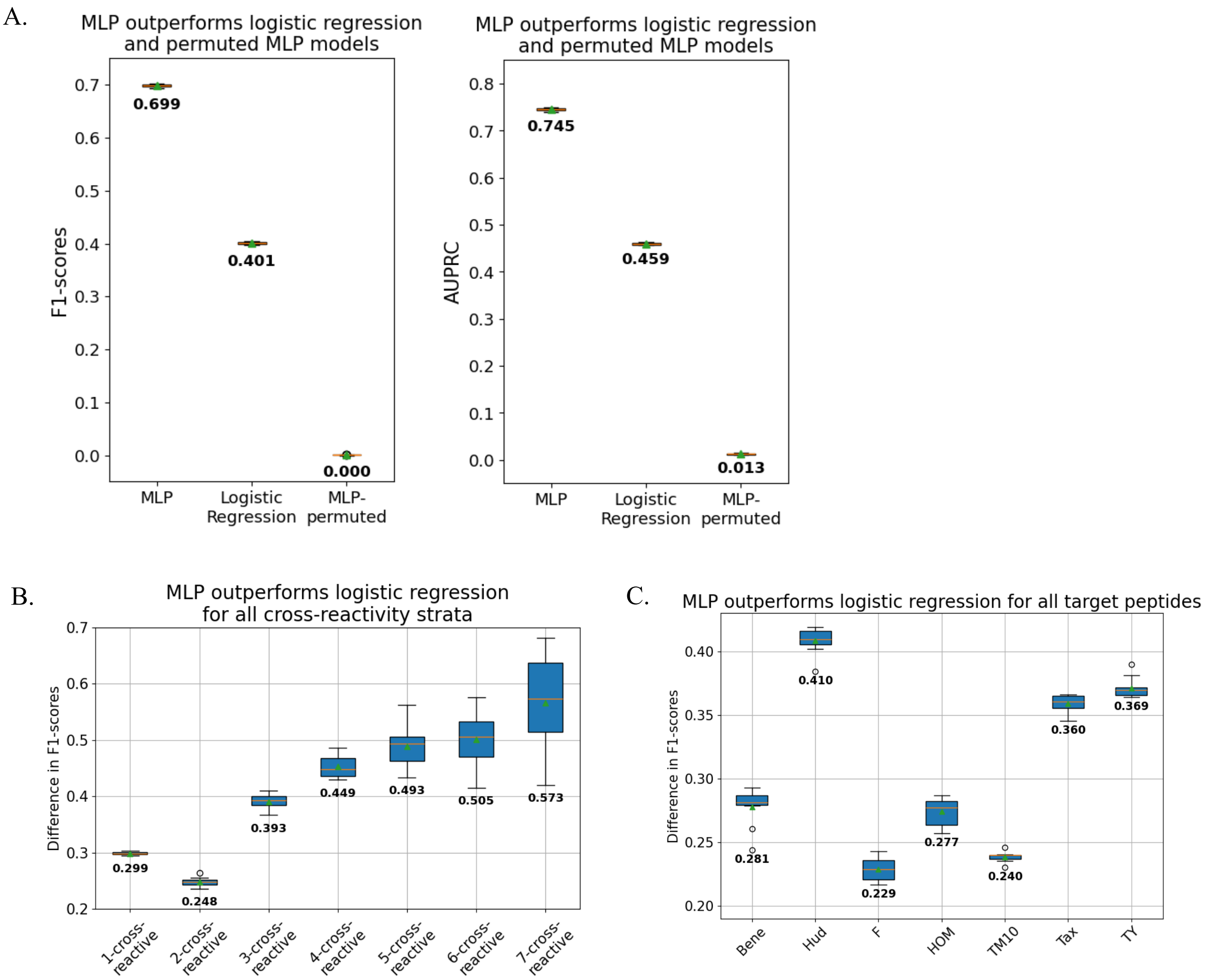

Extended Data Figure 4. VHSE8 embedding improves TCR binding classification, albeit to a less degree than BLOSUM50

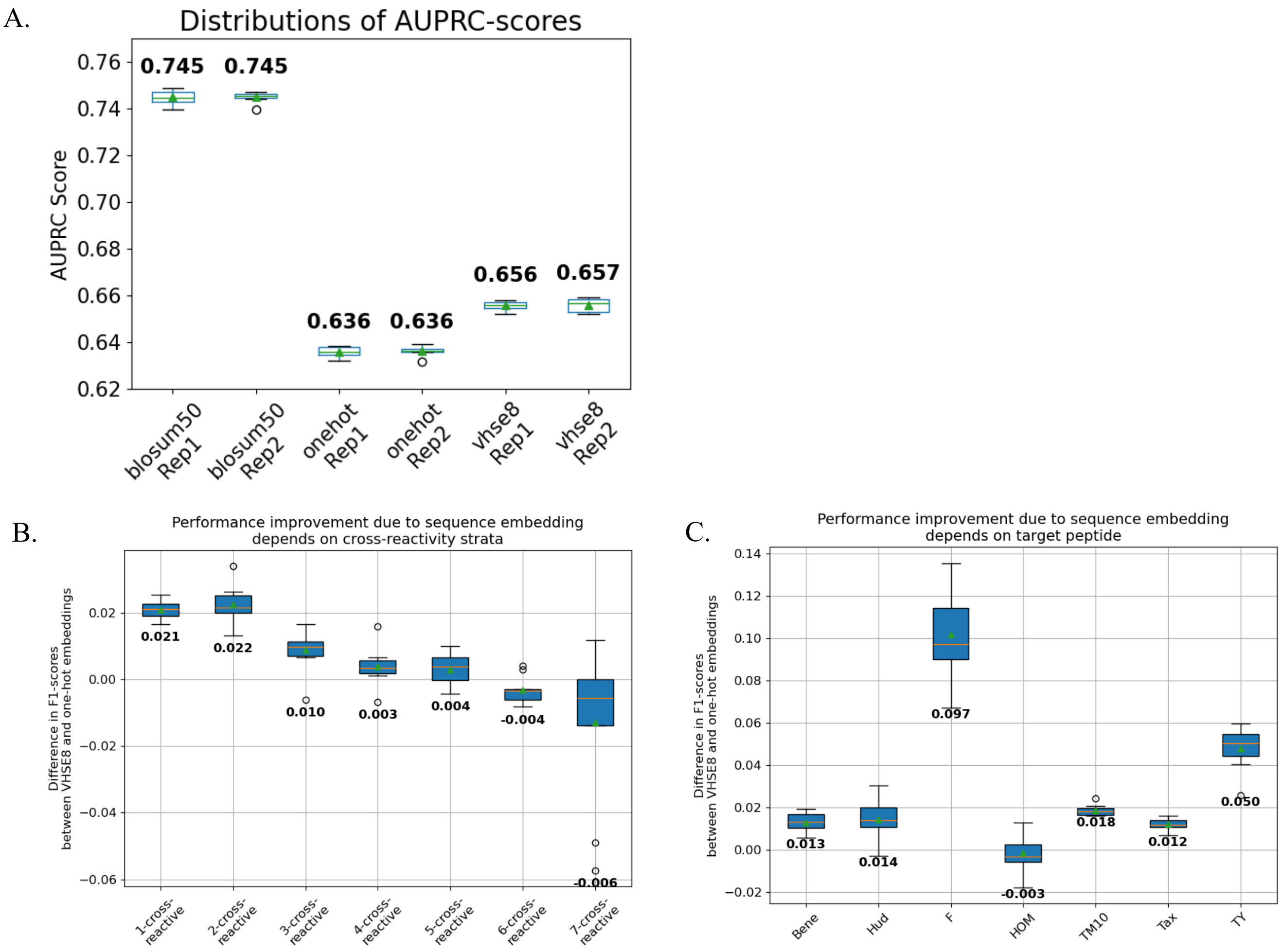

Extended Data Figure 5. Observed hierarchy of examined amino acid positions are invariant to the choices of sequence embeddings and performance metrics

A.

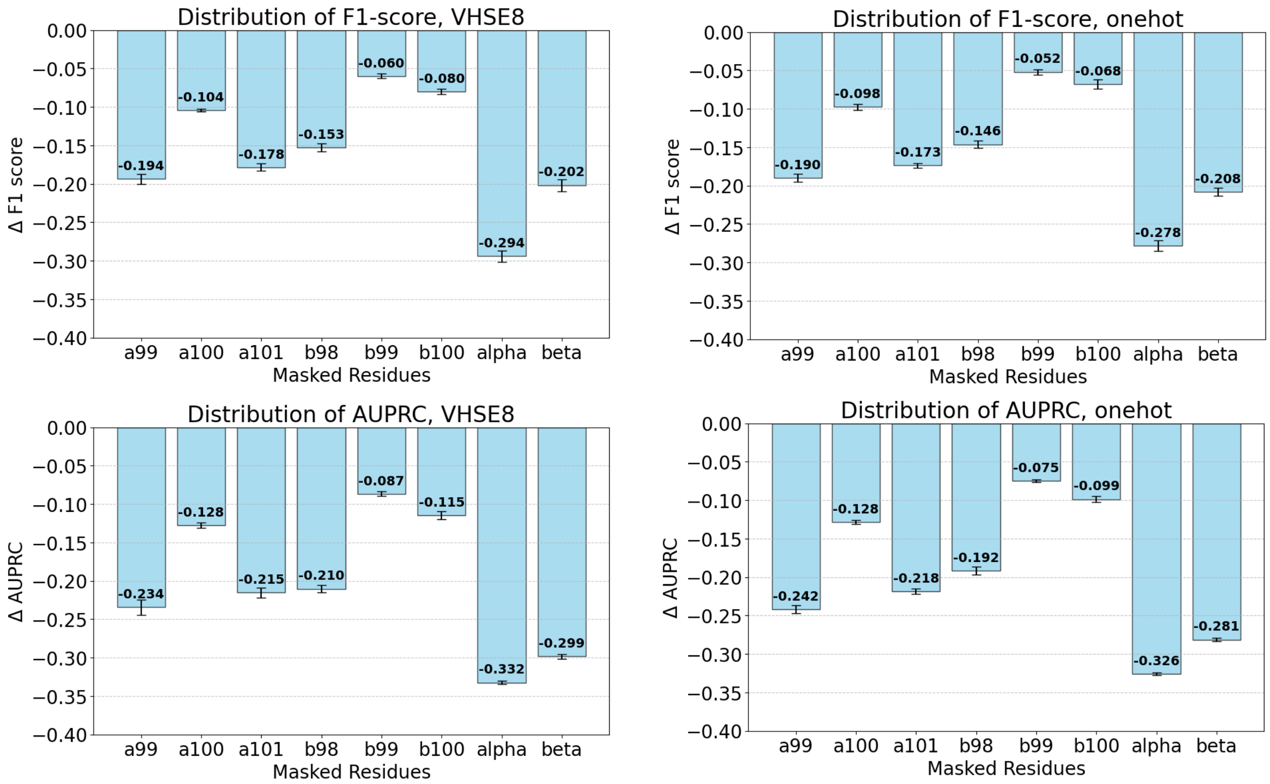

B.

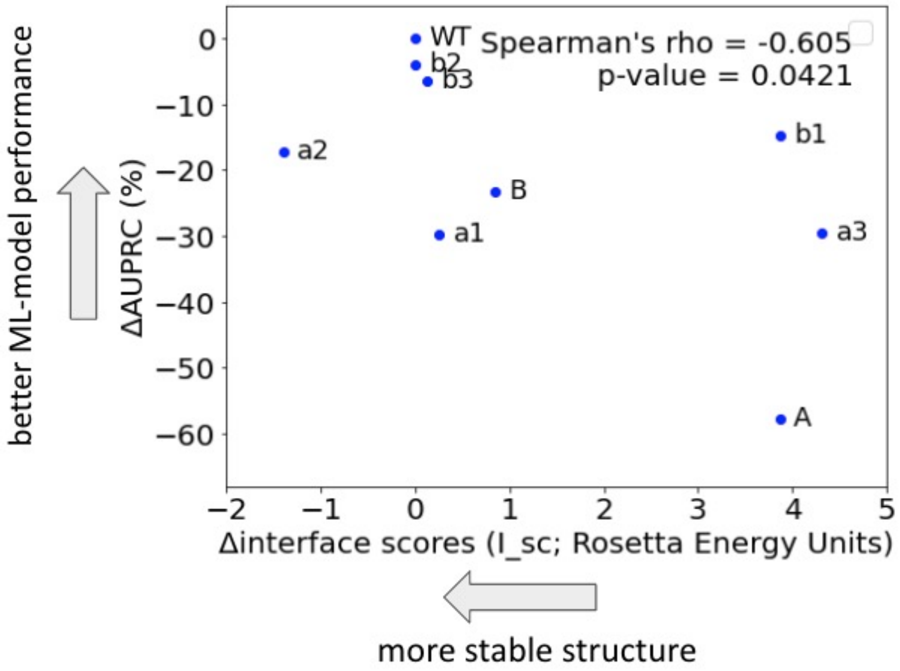

Extended Data Figure 6. BLOSUM-50 (BLOcks SUbstitution Matrix) matrix of amino acids.

	A	C	D	E	F	G	H	I	K	L	M	N	P	Q	R	S	T	V	W	Y
A	**5**	-1	-2	-1	-3	0	-2	-1	-1	-2	-1	-1	-1	-1	-2	1	0	0	-3	-2
C	-1	**13**	-4	-3	-2	-3	-3	-2	-3	-2	-2	-2	-4	-3	-4	-1	-1	-1	-5	-3
D	-2	-4	**8**	2	-5	-1	-1	-4	-1	-4	-4	2	-1	0	-2	0	-1	-4	-5	-3
E	-1	-3	2	**6**	-3	-3	0	-4	1	-3	-2	0	-1	2	0	-1	-1	-3	-3	-2
F	-3	-2	-5	-3	**8**	-4	-1	0	-4	1	0	-4	-4	-4	-3	-3	-2	-1	1	4
G	0	-3	-1	-3	-4	**8**	-2	-4	-2	-4	-3	0	-2	-2	-3	0	-2	-4	-3	-3
H	-2	-3	-1	0	-1	-2	**10**	-4	0	-3	-1	1	-2	1	0	-1	-2	-4	-3	2
I	-1	-2	-4	-4	0	-4	-4	**5**	-3	2	2	-3	-3	-3	-4	-3	-1	4	-3	-1
K	-1	-3	-1	1	-4	-2	0	-3	**6**	-3	-2	0	-1	2	3	0	-1	-3	-3	-2
L	-2	-2	-4	-3	1	-4	-3	2	-3	**5**	3	-4	-4	-2	-3	-3	-1	1	-2	-1
M	-1	-2	-4	-2	0	-3	-1	2	-2	3	**7**	-2	-3	0	-2	-2	-1	1	-1	0
N	-1	-2	2	0	-4	0	1	-3	0	-4	-2	**7**	-2	0	-1	1	0	-3	-4	-2
P	-1	-4	-1	-1	-4	-2	-2	-3	-1	-4	-3	-2	**10**	-1	-3	-1	-1	-3	-4	-3
Q	-1	-3	0	2	-4	-2	1	-3	2	-2	0	0	-1	**7**	1	0	-1	-3	-1	-1
R	-2	-4	-2	0	-3	-3	0	-4	3	-3	-2	-1	-3	1	**7**	-1	-1	-3	-3	-1
S	1	-1	0	-1	-3	0	-1	-3	0	-3	-2	1	-1	0	-1	**5**	2	-2	-4	-2
T	0	-1	-1	-1	-2	-2	-2	-1	-1	-1	-1	0	-1	-1	-1	2	**5**	0	-3	-2
V	0	-1	-4	-3	-1	-4	-4	4	-3	1	1	-3	-3	-3	-3	-2	0	**5**	-3	-1
W	-3	-5	-5	-3	1	-3	-3	-3	-3	-2	-1	-4	-4	-1	-3	-4	-3	-3	**15**	2
Y	-2	-3	-3	-2	4	-3	2	-1	-2	-1	0	-2	-3	-1	-1	-2	-2	-1	2	**8**

Extended Data Figure 7. VHSE-8 (Vectors of Hydrophobic, Steric, and Electronic properties-8) matrix of amino acids.

Amino Acids	$VHSE_1$	$VHSE_2$	$VHSE_3$	$VHSE_4$	$VHSE_5$	$VHSE_6$	$VHSE_7$	$VHSE_8$
Ala A	0.15	−1.11	−1.35	−0.92	0.02	−0.91	0.36	−0.48
Arg R	−1.47	1.45	1.24	1.27	1.55	1.47	1.30	0.83
Asn N	−0.99	0.00	−0.37	0.69	−0.55	0.85	0.73	−0.80
Asp D	−1.15	0.67	−0.41	−0.01	−2.68	1.31	0.03	0.56
Cys C	0.18	−1.67	−0.46	−0.21	0.00	1.20	−1.61	−0.19
Gln Q	−0.96	0.12	0.18	0.16	0.09	0.42	−0.20	−0.41
Glu E	−1.18	0.40	0.10	0.36	−2.16	−0.17	0.91	0.02
Gly G	−0.20	−1.53	−2.63	2.28	−0.53	−1.18	2.01	−1.34
His H	−0.43	−0.25	0.37	0.19	0.51	1.28	0.93	0.65
Ile I	1.27	−0.14	0.30	−1.80	0.30	−1.61	−0.16	−0.13
Leu L	1.36	0.07	0.26	−0.80	0.22	−1.37	0.08	−0.62
Lys K	−1.17	0.70	0.70	0.80	1.64	0.67	1.63	0.13
Met M	1.01	−0.53	0.43	0.00	0.23	0.10	−0.86	−0.68
Phe F	1.52	0.61	0.96	−0.16	0.25	0.28	−1.33	−0.20
Pro P	0.22	−0.17	−0.50	0.05	−0.01	−1.34	−0.19	3.56
Ser S	−0.67	−0.86	−1.07	−0.41	−0.32	0.27	−0.64	0.11
Thr T	−0.34	−0.51	−0.55	−1.06	−0.06	−0.01	−0.79	0.39
Trp W	1.50	2.06	1.79	0.75	0.75	−0.13	−1.01	−0.85
Tyr Y	0.61	1.60	1.17	0.73	0.53	0.25	−0.96	−0.52
Val V	0.76	−0.92	−0.17	−1.91	0.22	−1.40	−0.24	−0.03
